# Wrist-ankle acupuncture has a positive effect on cancer pain: a meta-analysis

**DOI:** 10.1186/s12906-020-03193-y

**Published:** 2021-01-07

**Authors:** Bei Dong, Lu Lin, Qiuyun Chen, Yishu Qi, Fen Wang, Keyan Qian, Li Tian

**Affiliations:** 1 Oncology Department of the First Affiliated Hospital of Soochow University, No. 188 Shizi Road, Suzhou, 215006 China; 2 School of Nursing, Medical College of Soochow University, No. 188 Shizi Road, Suzhou, 215006 China; 3 Radiotherapy Department of the First Affiliated Hospital of Soochow University, Suzhou, China

**Keywords:** Wrist-ankle acupuncture, Pain, Cancer, Meta-analysis, Randomized controlled trial

## Abstract

**Background:**

Wrist-ankle acupuncture (WAA) as a kind of micro acupuncture therapy has been used to management cancer pain, however, the effects of WAA on cancer pain were controversial in the current studies. Therefore, the purpose of this meta-analysis was to critically evaluate the effect of wrist-ankle acupuncture (WAA) on cancer pain.

**Methods:**

Seven digital databases were searched from the inception of databases to July 2020, including CNKI, Wanfang, VIP, CBM, Cochrane Library, PubMed and Embase. Randomized controlled trials conforming to the inclusion and exclusion criteria were screened and extracted; the risk of bias was evaluated using the Cochrane Collaboration criteria. The primary outcome indicators included pain relief rate and pain score, and the secondary outcome was adverse reaction incidence. All analyses were performed with Review Manager 5.3.

**Results:**

A total of 13 studies with 1005 cancer patients (intervention group: 568, control group: 437) were included in this meta-analysis. The results demonstrated that the pain relief rate of experimental group (WAA / WAA + drug intervention) was better than that of control group (analgesic drug intervention), and the difference was statistically significant [RR = 1.31, 95%CI: 1.15 ~ 1.49, *P* < 0.01].

**Conclusions:**

WAA has certain effect on cancer pain, and the effect of WAA combined with pharmacological intervention is better than that of drug therapy alone.

## Background

Cancer pain is caused by cancer itself or treatment and psychological factors, which is long-term and lasting [1, 2]. A study has revealed that 40% of early-stage cancer patients and 90% of advanced cancer patients experience moderate or severe pain, and 70% of the patients do not get sufficient pain relief [3]. Pain can interfere with daily activities such as sleep, mood and social intercourse, and seriously affect the quality of life of patients [4, 5]. Cancer pain is mainly controlled by opioids, and the commonly used drugs are oxycodone, morphine, fentanyl transdermal patch and codeine [6]. However, drug therapy has obvious toxic and side effects, and patients are prone to constipation, vomiting, urinary retention, delirium, dizziness, and other adverse reactions [7]. Additionally, long-term drug analgesia tends to increase the dosage of analgesics since patients will develop drug tolerance over time, which will aggravate the economic burden for patients due to high drug prices [8, 9]. Therefore, non- pharmacological therapies for cancer pain are attracting more and more attention.

Non-pharmacological intervention of cancer pain mainly includes psychological education intervention, cognitive behavior intervention, complementary and alternative medicine, and comprehensive non-pharmacological intervention [10]. Acupuncture is one of the complementary and alternative therapies. Acupuncture for analgesia has a long history in China and its use has been widely recognized and accepted [11]. Among them, wrist-ankle acupuncture (WAA) is a kind of micro acupuncture therapy invented by Professor Zhang Xinshu [12] of the Second Military Medical University in the 1970s. It is based on electrical stimulation therapy and combined with modern neurology theory and traditional acupuncture theory [9]. The acupuncture site of WAA is limited to the wrist and ankle, but the treatment range is all over the body, which features simple operation and high safety. Current clinical studies have indicated that WAA has significant efficacy in orthopedic pain, dysmenorrhea, soft tissue pain, toothache and so on [9, 13]. Zhou’s research demonstrated that WAA can increase serotonin levels in the brain, and raise the pain threshold to achieve pain relief [14]; and Chen’s study found that the analgesic effect of WAA may be associated with promoting the release of β-endorphins in plasma and inhibiting the production of substance P [15].

In recent years, studies comparing the effect of WAA on cancer pain with drug therapy have been increased significantly. Some studies showed that WAA or WAA plus pharmacological intervention was more effective while others had opposite results. Therefore, the purpose of this meta-analysis was to critically assess the effect of WAA on cancer pain so as to provide scientific reference for the development of intervention strategy for cancer pain.

## Methods

This meta-analysis was performed following the PRISMA guidelines for systematic reviews and meta-analyses [16].

### Searching strategies

This study systematically searched seven digital databases, which were China National Knowledge Infrastructure (CNKI), Wanfang, VIP, China Biology Medicine (CBM), Cochrane Library, PubMed and Embase from the inception of databases to July 2020 for randomized controlled trials (RCTs) without language restrictions. Two researchers independently read the title, abstract and full text to screen the studies that could be included in the meta-analysis. If there was any dispute, a third person was asked to reach a consensus. The search strategies for the English databases are shown in Additional file 1.

### Inclusion criteria

#### Participants

Studies including adult patients (≥18 years) who were diagnosed with cancer and suffering from pain, regardless of cancer stage and current treatment, were eligible.

#### Interventions and controls

The intervention was wrist-ankle acupuncture alone or wrist-ankle acupuncture plus analgesics, while the control group was intervened with analgesics (drug types were not limited). The intervention group and the control group had the same drug intervention.

#### Outcomes

The outcome indicators were pain relief rate, pain score, and adverse reaction rate. The analgesic effects of the interventions were classified into four levels: (i) Complete Remission (CR): completely pain-free; (ii) Partial Remission (PR): substantial relief of pain and generally normal sleep; (iii) Mild Remission (MR): moderate relief of pain with residual pain and sleep disturbance; (iv) No remission (NR): no relief of pain. The pain relief rate was based on the significant effective rate (n _(CR + PR)_ / *n* * 100%) [17].

#### Types of studies

Only RCTs were eligible.

### Data extraction

The data were extracted and cross-checked by two researchers independently. In case of differences, a third party would be asked to judge. The basic data extracted mainly included the first author of the literature, year of publication, number of participants in the intervention group and the control group, age of participants, tumor type, intervention and control measures, indicators of effect evaluation and incidence of adverse reaction. If the key information was missing, the authors of the report were contacted to obtain the information.

### Risk of bias assessment

Two reviewers independently evaluated the risk of bias using the Cochrane assessment tool, which consists of the following seven domains: “adequate sequence generation, allocation concealment, blinding of participants and personnel, blinding of outcome assessment, incomplete outcome data, selective reporting, and other bias” [16]. Each question can be rated as follows: yes (+), low risk of bias; unclear (?), unclear risk of bias; no (−), high risk of bias.

### Data analysis

Review Manager 5.3 software was used for statistical analysis. Risk ratio (*RR*) was used for enumeration data, and standardized mean difference (*SMD*) was used for continuous data. Reporting and publication biases of the included studies were assessed by visually inspecting the asymmetry of the funnel plot. In each analysis, *I*^*2*^ was used to measure the statistical heterogeneity among the trials. If *P* > 0.1 and *I*^*2*^ < 50%, due to the homogeneity of the trials, the fixed effects model was used for analysis; if *P* < 0.1 and *I*^*2*^ ≥ 50%, the random effects model was used. If *P* < 0.1 and the source of heterogeneity was unidentified, a descriptive analysis was performed instead of a meta-analysis [16]. If moderate clinical heterogeneity was found, a subgroup analysis was conducted. Sensitivity analysis was used to explore the effects of the fixed effects model and random effects model analyses on heterogeneity results and the effects of any assumptions [18].

## Results

### Literature search

**Fig. 1:**
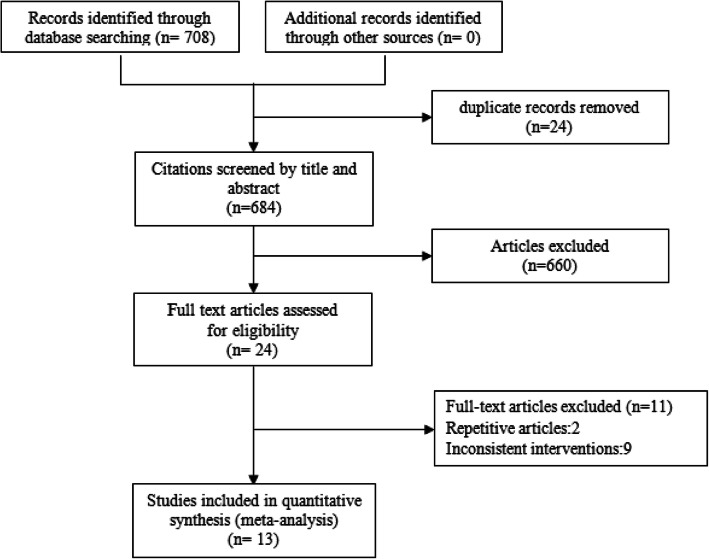
Flow chart diagram of trial identification and selection

Seven hundred eighth records in total were identified through database searching, of which 24 were duplicates. After a preliminary review of the titles and abstracts, 660 records were excluded for not meeting the inclusion criteria. Among the remaining 24 trials, 9 were excluded because the intervention measures used did not conform to the requirements, and 2 articles were duplicates. Therefore, a total of 13 trials were included [19–31], with a total of 1005 patients. The process of trial identification and selection is shown in Fig. 1.

### Characteristics of the included studies

The general information of the literature included is shown in Table 1. The number of eligible studies published in 2018 and 2019 is largest, 3 articles a year. All the WAA interventions were conducted in China. The age of the participants ranged from 19 to 81 years; the participants were mainly male, and only one study had more than 50% female participants in the intervention group while other studies had less than 50% (except for those not reported). Among the included studies, seven studies were concerned with comprehensive cancer types; four were on hepatic carcinoma, and the remaining two were on gastric cancer and bone metastasis of prostate cancer. WAA intervention alone was reported in 6 studies, while WAA plus drug therapy was used in 10 studies. The specific characteristics of the included WAA intervention studies are shown in Table 2.

**Table 1:** Studies included in the systematic review

Author, year	Sample	Age	Gender	Cancer type	The intervention for treatment group	The intervention for control group	Outcome indicator	Adverse reactions (%)
Shen, 2000 [19]	ADG: 34 CG: 31	ADG: 24–76 CG: 32–68	ADG:female 3 male 31 CG: female 3 male 28	Mixed	WAA + drug	Grade II pain: weak opioid analgesics plus adjuvants Grade III pain: strong opioid analgesics plus adjuvants	Pain relief rate	Not reported
Hu, 2004 [20]	Moderate pain group AG: 20 CG I: 20 Severe pain group AG: 16 CG II: 20 ADG: 18	Moderate pain group AG:48.9 ± 13.0 CG I:49.7 ± 13.5 Severe pain group AG: 48.3 ± 7.9 CG II:51.1 ± 8.9 ADG:53.6 ± 11.3	Moderate pain group AG:female 2 male 18 CG I:female 3 male 17 Severe pain group AG: female 2 male 14 CG II:female 3 male 17 ADG:female 0 male 18	Liver cancer	WAA/ WAA + drug	CG I: weak opioids (Such as codeine) CGII: morphine sulfate sustained-release tablets as a representative treatment	Pain relief rate; NRS	ADG: 3(16.7) CG II: 12(60)
Han, 2012 [21]	AG:25 CG:25	AG:20–71 CG:19–73	AG:female 8 male 17 CG:female 6 male 19	Liver cancer	WAA	Three-step drug analgesia	Pain relief rate	AG: 2 (8) CG: 19 (76)
Author, year	Sample	Age	Gender	Cancer type	The intervention for treatment group	The intervention for control group	Outcome indicator	Adverse reactions (%)
Zeng, 2014 [22]	AG:30 CG:30	AG:53.8 ± 12.8 CG:49.0 ± 14.1	AG:female 3 male 27 CG:female 2 male 28	Liver cancer	WAA	A single dose of morphine sulfate 10 mg	Pain relief rate	Unclear
Dong, 2015 [23]	AG:49 CG:36 ADG:41	AG:61 ± 13 CG:59 ± 12 ADG:63 ± 11	AG:female 16 male 33 CG:female 13 male 23 ADG:female 14 male 27	Liver cancer	WAA / WAA + drug	Three-step drug analgesia	Pain relief rate	AG:12(24.5) CG:35(97.2) ADG:39(95.1)
Wang, 2017 [24]	AG:20 CG:20 ADG:20	19–81	Female 25 male 35	Mixed	WAA / WAA + drug	Three-step drug analgesia	Pain relief rate	AG: 2 (10) CG: 12 (60) ADG: 6 (30)
Dong, 2018 [25]	ADG: 60 CG: 60	47.65 ± 22.41	Female 41 male 79	Mixed	WAA + drug	Strong opioid analgesia	Pain relief rate; NRS	ADG:35(58.3) CG: 45 (75)
Zhang, 2018 [26]	ADG: 76 CG: 34	ADG: 46–69 CG: 45–75	ADG: female 26 male 50 CG: female 13 male 21	Gastric cancer	WAA + drug	Drug therapy	Pain relief rate	Not reported
Su, 2018 [27]	AG: 40 CG: 40	76.6 ± 2.5	Not reported	Bone metasta- sis of prostate cancer	WAA	Drug therapy	VAS	2(2.5)
Author, year	Sample	Age	Gender	Cancer type	The intervention for treatment group	The intervention for control group	Outcome indicator	Adverse reactions (%)
Fu, 2019 [28]	ADG: 18 CG: 18	ADG: 66 ± 12 CG: 64 ± 15	ADG: female 8 male 10 CG: female 8 male 10	Mixed	WAA + drug	Morphine sustained release tablets	Pain relief rate; NRS	ADG:3(18.75) CG: 9(56.25)
Luan, 2019 [29]	ADG: 32 CG: 33	ADG: 31–70 CG: 44–68	ADG:female 15 male 17 CG: female 13 male 20	Mixed	WAA + drug	Opioid analgesics	Pain relief rate	Not reported
Wu, 2019 [30]	ADG: 30 CG: 30	ADG: 56 ± 3 CG: 58 ± 3	ADG:female 15 male 15 CG: female 14 male 16	Mixed	WAA + drug	Three-step drug analgesia	Pain relief rate; VAS	≥ grade III: ADG: 0(0) CG: 6(20)
Xu, 2020 [31]	ADG: 39 CG: 40	ADG:64.9 ± 11.0 CG:67.33 ± 13.1	ADG:female 21 male 18 CG: female 16 male 24	Mixed	WAA+ drug	Three-step drug analgesia	Pain relief rate	Not reported

*Note*: *AG* Acupuncture Group (The acupuncture intervention is wrist-ankle acupuncture), *ADG* Acupuncture + Drug Group (The acupuncture intervention is wrist-ankle acupuncture; The drug treatment is the same as the control group), *CG* Control Group, *WAA* Wrist-Ankle Acupuncture, *NRS* Numerical Rating Scale, *VAS* Visual Analogue Scale

**Table 2:** The characteristics of wrist-ankle acupuncture intervention

Author, year	Needle specifications; angles of needling insertion; exposed length of needle body; needle retention time; course of treatment
Shen, 2000 [19]	Not reported; 15°; 1 cm; 24–72 h; 10 days/course, 2 courses
Hu, 2004 [20]	0.25 mm × 250 mm; 30°; not reported; 10–12 h; 10 days/course, 1 course
Han, 2012 [21]	0.25 mm × 250 mm; 30°; not reported; 10–12 h; 10 days/course, 1 course
Zeng, 2014 [22]	0.25 mm × 250 mm; 30°; not reported; 6 h; 1 time treatment
Dong, 2015 [23]	0.25 mm × 250 mm; 30°; 2 mm; 10 h; not reported
Wang, 2017 [24]	Not reported; 30°; 1 mm; 12 h; 10 day/course, 3 courses
Dong, 2018 [25]	0.25 mm × 250 mm; 30°; 1 mm; 10–12 h; 7 days/course
Zhang, 2018 [26]	0.25 mm × 250 mm; 30°; not reported; 1–2 h; 14 days/course, 1 course
Su, 2018 [27]	0.25 mm × 250 mm; not reported; not reported; 9–12 h; 10 days/course, 1 course
Fu, 2019 [28]	0.25 mm × 250 mm; 30°; not reported; 1 h; not reported
Luan, 2019 [29]	0.3 mm × 25 mm; 30°; 2 mm; 1 h; not reported
Wu, 2019 [30]	0.2 mm × 25 mm; 20–30°; 1 mm; 12 h; 10 days/course, 1 course
Xu, 2020 [31]	0.3 mm × 25 mm; not reported; 0 mm; 2–12 h; not reported

### Risk of bias in individual trial

**Fig. 2:**
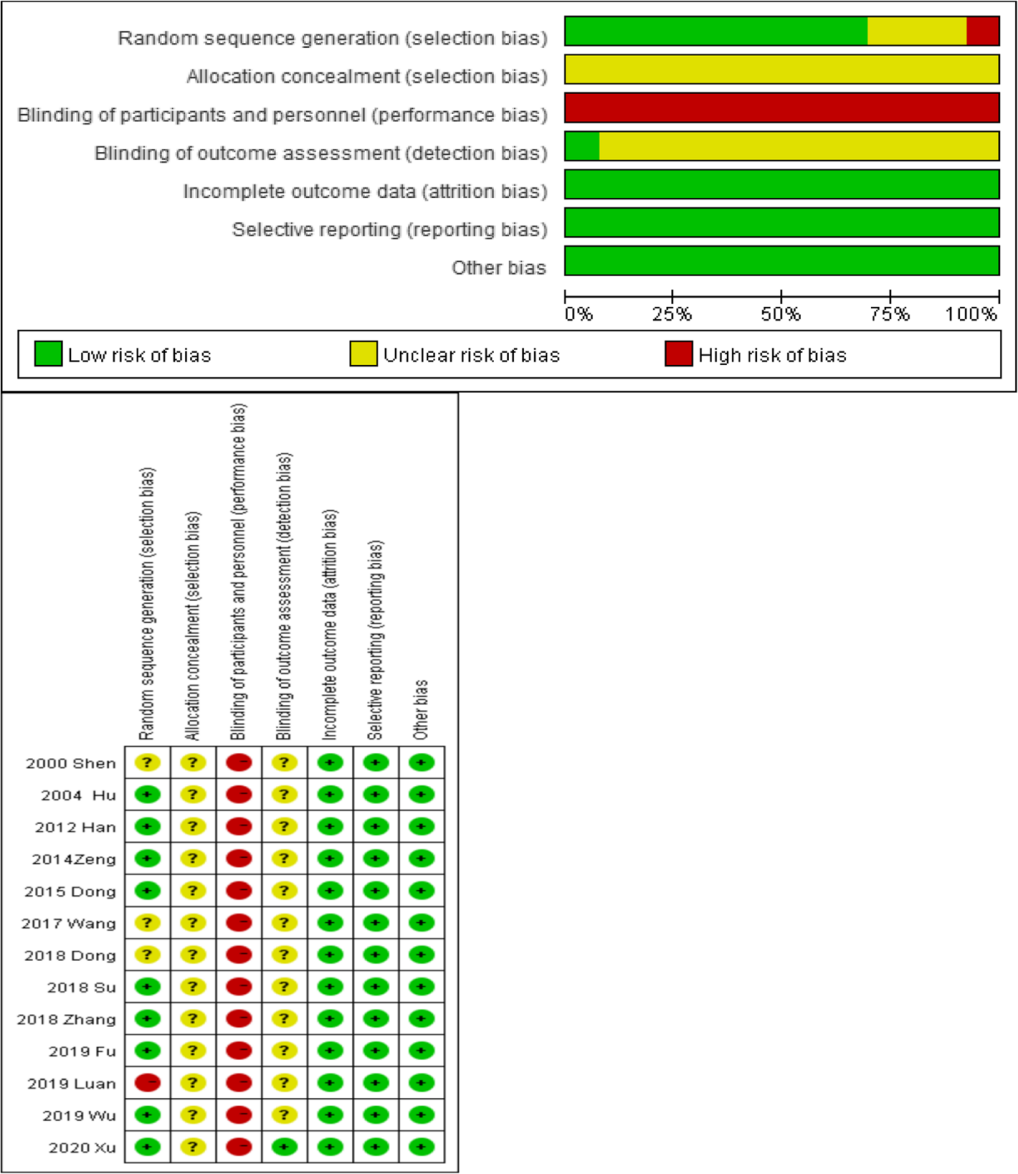
Risk of bias assessment using the Cochrane tool

The risk of bias as shown in Fig. 2 was moderate; blinding of participants and personnel was not applicable for WAA intervention, so the risk of performance bias was high in all studies. Ten studies reported the random sequence generation [20–23, 26–31], one of which was high risk [29]. However, no studies had reported the allocation concealment, and only 1 study reported blinding of outcome assessment and had a low risk [31].

### Analysis of overall effects

#### Pain relief rate

**Fig. 3:**
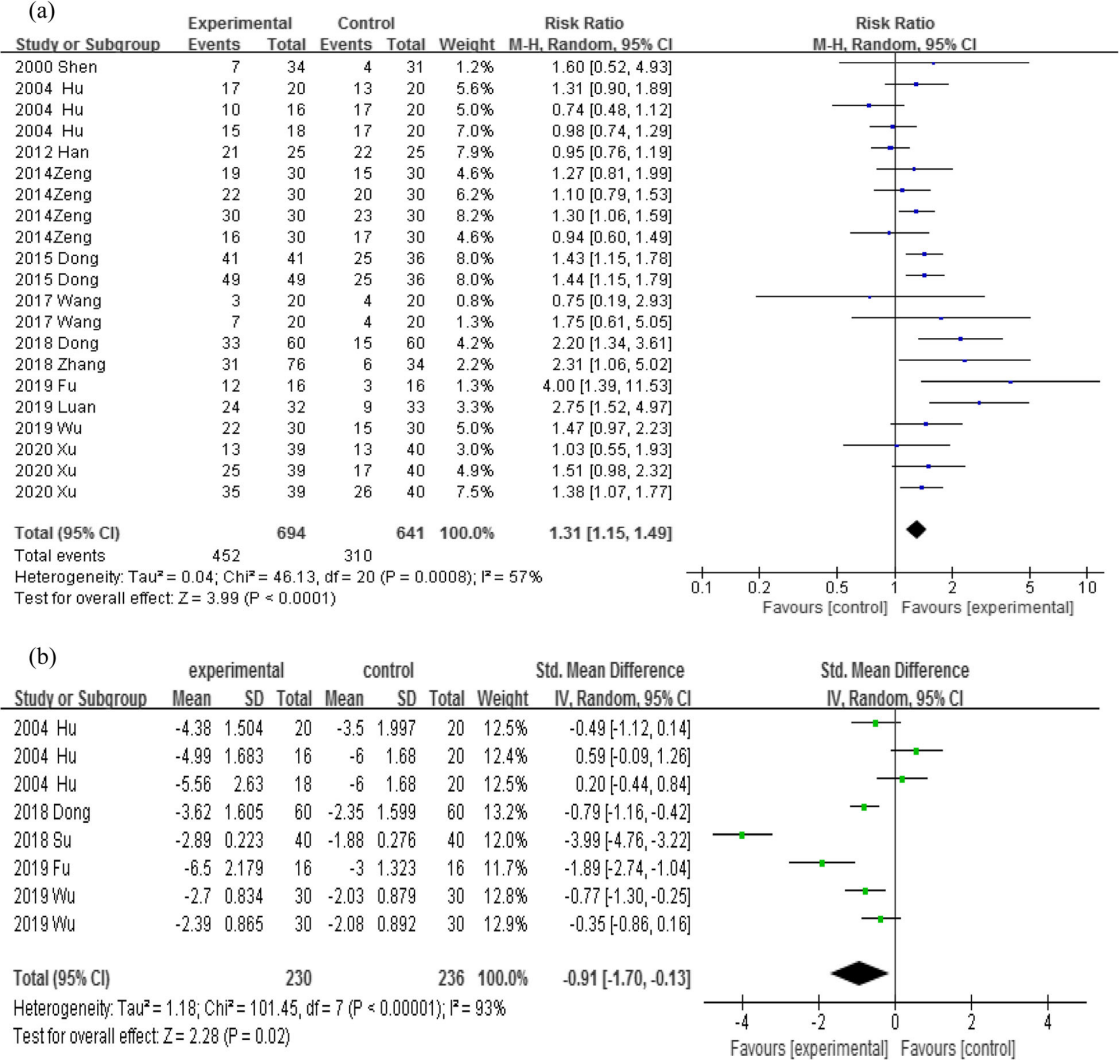
Forest plots of WAA/ WAA plus drug therapy versus drug therapy: **a** Pain relief rate; **b** Pain score

**Fig. 4:**
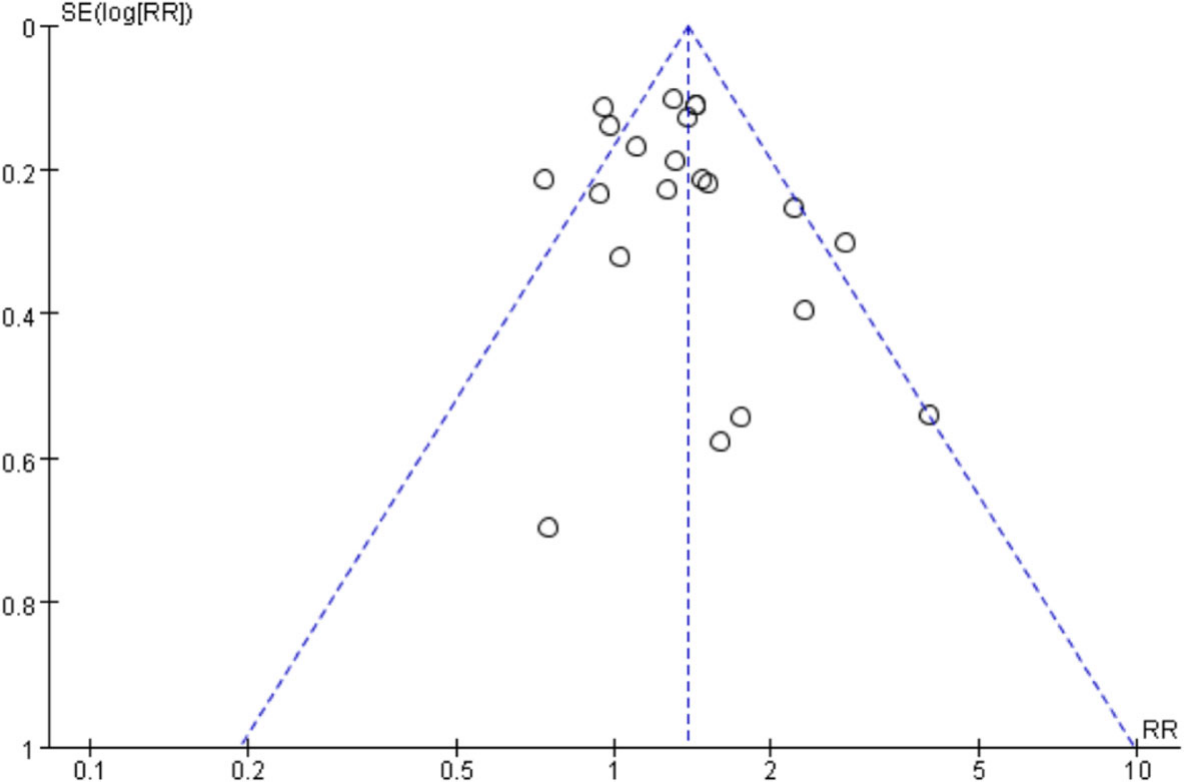
Funnel plots of pain relief rates

Twelve articles [19–26, 28–31] reported pain relief rates of the intervention group and control group. The results demonstrated that the pain relief rate in the intervention group (including WAA group and WAA plus drug therapy group) was significantly higher than that in the control group (drug therapy group) [*RR* = 1.31, *95%CI*: 1.15–1.49, *P* < 0.01]. The differences between the two groups in pain relief rate or pain score are shown in Fig. 3. The funnel plot (Fig. 4) indicated that the publication bias was mild, and the sensitivity analysis [*RR* = 1.38, *95%CI*: 1.26 ~ 1.50, *P* < 0.01] revealed that the model was relatively stable.

The subgroup analysis was performed based on whether WAA was combined with analgesic drug therapy. The outcomes indicated that both the WAA alone group and the control group (using analgesics) had pain remission after intervention, but there was no statistical difference in pain relief rate between the two groups [*RR* = 1.13, *95%CI*: 0.98 ~ 1.32, *P* = 0.09]. The pain relief rate of WAA plus drug therapy group was significantly higher than that of control group (using analgesics) [*RR* = 1.55, *95% CI*: 1.26–1.91, *P* = 0.01]. The results of sensitivity analysis are displayed in Table 3.

**Table 3:** Results of subgroup analysis and sensitivity analysis of pain relief rate

Pain relief rate	Sample size		Random-effects analysis				Fixed-effects analysis			
	TG	CG	RR	95%CI		*P*	RR	95%CI		*P*
				L	U			L	U	
Type of intervention										
WAA	250	241	1.13	0.98	1.32	0.09	1.14	1.02	1.28	0.02
WAA + drug	444	400	1.55	1.26	1.91	0.01	1.61	1.41	1.85	< 0.01

*Note*: *TG* Treatment group, *CG* Control group, *RR* Risk Ratio, *L* Lower, *U* Upper

#### Pain score

Five studies [20, 25, 27, 28, 30] reported the pain score before and after intervention. The pain scores of the intervention group and control group were both decreased after trials, and there were statistically significant differences in the scores within the group. The results of the meta-analyses demonstrated that there was a statistical difference in pain score between the intervention group and control group [*SMD* = − 0.91, *95%CI*: − 1.70 ~ − 0.13, *P* = 0.02] (Fig. 3), and the sensitivity analysis also indicated statistically significant differences [*SMD* = − 0.75, *95%CI*: − 0.95 ~ − 0.55, *P* < 0.01].

#### Adverse reactions rate

Seven studies [20, 21, 23–25, 28, 30] reported the adverse reactions rate clearly, with that of the intervention group being significantly lower than that of the control group (16.7% VS 60%; 8% VS 76%; 24.5%/ 95.1% VS 97.2%; 10%/ 30% VS 60%; 58.3% VS 75%; 18.75% VS 56.25%; 0% VS 20%). The main adverse reactions of WAA were subcutaneous hemorrhage and dizziness, and those of drug therapy were dizziness, nausea, vomiting, drowsiness, constipation, and urinary retention.

## Discussion

This meta-analysis included 13 medium-quality studies conducted on a total of 1005 participants. All studies did not report allocation concealment, and only one study reported blinding of outcome assessment. Seven studies were on mixed cancer types; four were on liver cancer and the remaining two were on gastric cancer and prostate cancer with bone metastasis.

The meta-analysis of pain relief rate showed that the pain relief rate in the intervention group (WAA/WAA plus drug therapy group) was significantly higher than that in the control group (drug therapy group). Simultaneously, the meta-analysis of pain score also demonstrated that the intervention group had statistically significant effect compared with the control group. However, the results of meta-analysis by Zheng Yi et al. [32] in 2014 indicated that there was no statistical difference between the intervention group (WAA/WAA plus drug therapy group) and the control group (drug therapy group) in pain relief rate, which may be related to the limited number and poor quality of included studies. In this study, more RCTs with higher quality published in recent 3 years have been included, and thus the results have higher reliability and acceptability.

Subgroup analysis was conducted based on whether WAA was combined with drug therapy, of which the results showed that there was no statistical difference in pain relief rate between the intervention group (WAA) and control group (using analgesics). However, the sensitivity analysis demonstrated that the difference was statistically significant, indicating that the results were unstable. The sample size of some included studies was too small [20, 21, 24]_,_ which may have influence on the results, so the results of this meta-analysis should be treated with caution. The pain relief rate of the WAA plus drug therapy group was higher than that of the control group (using analgesics), and the sensitivity analysis also showed the same results, indicating that the results were reliable. Therefore, more large-sample studies are needed to verify the difference in effect between WAA alone and drug therapy.

All the intervention groups (WAA/WAA plus drug therapy group) included in this study had a statistically significant pain relief. Meanwhile, WAA had faster and longer analgesic effect, so the number of pain outbreaks can be reduced effectively [20, 22, 23, 25, 28, 30]. Moreover, dosage reduction and drug withdrawal were also observed in the WAA plus drug therapy group [19, 20, 28, 31]. Seven studies reported the adverse reactions rate clearly, and that of the intervention group was significantly lower than that of the control group [20, 21, 23–25, 28, 30]. These findings indicate that WAA could relieve cancer pain better, reduce the dependence on analgesics, has fewer side effects, and therefore it is an economical and effective treatment for cancer pain.

### Limitations

Despite our comprehensive review of the literature on using WAA to treat cancer pain in cancer patients, the present study still has some limitations. First, the quality of the studies included in this meta-analysis is mediocre, and the report of sequence generation and allocation concealment is incomplete. Second, most included studies were written in Chinese, and only 2 English articles met the inclusion criteria, which has certain selection bias. Third, we were unable to conduct subgroup analysis on cancer types and explore the effect of WAA on different types of cancer, because the cancer type of most studies was comprehensive and there were few studies on a single type of cancer.

## Conclusions

In conclusion, WAA has a certain effect on cancer pain. The analgesic effect of WAA plus drug therapy is better than drug therapy alone. Due to the medium risk of performance bias in the included studies, the findings of this meta-analysis should be interpreted with caution. Therefore, the efficacy of WAA alone still needs more larger-sample-size, high-quality and multi-center RCTs to verify in order to provide evidence for clinical treatment.

## Supplementary Information

**Additional file 1.**

## Data Availability

Data supporting our findings are contained within the manuscript.

## Abbreviations

WAA: Wrist-ankle acupuncture
CNKI: China National Knowledge Infrastructure
CBM: China Biology Medicine
RCTs: Randomized controlled trials
CR: Complete Remission
PR: Partial Remission
MR: Mild Remission
NR: No remission
RR: Risk ratio
SMD: Standardized mean difference
