# Supplementary material for: Wrist-ankle acupuncture has a positive effect on cancer pain: a meta-analysis
Source: BMC Complement Med Ther. 2021 Jan 7;21:24. doi: 10.1186/s12906-020-03193-y (PMC7791657; doi:10.1186/s12906-020-03193-y)
Supplement: Supplementary file 1 — Additional file 1. [file 12906_2020_3193_MOESM1_ESM.doc]

**Appendix Ⅰ**

| Database | Search strategy |
| --- | --- |
| Medline | #1 Randomized Controlled Trial [Publication Type] OR randomized [Title/Abstract] OR placebo [Title/Abstract] |
|  | #2 “Neoplasms”[Mesh] OR Neoplasia[Title/Abstract] OR Neoplasias [Title/Abstract] OR Neoplasm [Title/Abstract] OR Tumors[Title/Abstract] OR Tumor[Title/Abstract] OR Cancer[Title/Abstract] OR Cancers[Title/ Abstract] OR carcinoma[Title/Abstract] OR Malignancy[Title/Abstract] OR Malignancies[Title/Abstract] OR Malignant Neoplasms[Title/Abstract] OR Malignant Neoplasm[Title/Abstract] OR Neoplasm, Malignant[Title/Abstract] OR Neoplasms,Malignant[Title/Abstract] OR Benign Neoplasms[Title/Abstract] OR Neoplasms,Benign[Title/Abstract] OR Benign Neoplasm[Title/Abstract] OR Neoplasm,Benign[Title/ Abstract] |
|  | #3 “Pain”[Mesh] OR Pain, Burning[Title/Abstract] OR Burning Pain [Title/Abstract] OR Burning Pains[Title/Abstract] OR Pains,Burning[Title/Abstract] OR Suffering, Physical[Title/Abstract] OR Physical Suffering[Title/Abstract] OR Physical Sufferings[Title/Abstract] OR Sufferings,Physical[Title/Abstract] OR Pain,Migratory [Title/Abstract] OR Migratory Pain[Title/Abstract] OR Migratory Pains [Title/ Abstract] OR Pains,Migratory[Title/Abstract] OR Radiating Pain[Title/ Abstract] OR Pains, Radiating [Title/Abstract] OR Radiating Pain [Title/Abstract] OR Radiating Pains [Title/Abstract] OR Pain,Splitting[Title/Abstract] OR Pains,Splitting[Title/ Abstract] OR Splitting Pain[Title/Abstract] OR Splitting Pains[Title/Abstract] OR Ache[Title/Abstract] OR Aches[Title/Abstract] OR Pain,Crushing[Title/Abstract] OR Crushing Pain [Title/Abstract] OR Crushing Pains[Title/Abstract] OR Pains,Crushing [Title/Abstract] |
|  | #4 wrist-ankle acupuncture[Title/Abstract] OR wrist-ankle needle [Title/Abstract] |
|  | #5 # 1 AND #2 AND #3 AND #4 |
| Cochrane library | #1 (double-blind):ti,ab,kw OR (random):ti,ab,kw OR (control):ti,ab,kw OR (Randomized controlled trial)ti,ab,kw OR (randomized):ti,ab,kw OR (placebo): ti,ab,kw |
|  | #2 (Neoplasms):ti,ab,kw OR (Neoplasia):ti,ab,kw OR (Neoplasias):ti,ab,kw OR (Neoplasm):ti,ab,kw OR (Tumors):ti,ab,kw OR (Tumor):ti,ab,kw OR (Cancer): ti,ab,kw OR (Cancers):ti,ab,kw OR (Malignancy):ti,ab,kw OR (Malignancies): ti,ab,kw OR (Malignant Neoplasms):ti,ab,kw OR (Malignant Neoplasm):ti,ab,kw OR (Neoplasm,Malignant):ti,ab,kw OR (Neoplasms,Malignant):ti,ab,kw OR (Benign Neoplasms):ti,ab,kw OR (Neoplasms,Benign):ti,ab,kw OR (Benign Neoplasm): ti,ab,kw OR (Neoplasm, Benign):ti,ab,kw OR (carcinoma):ti,ab,kw |
|  | #3 (Pain):ti,ab,kw OR (Pain,Burning):ti,ab,kw OR (Burning Pain): ti,ab,kw OR (Burning Pains):ti,ab,kw OR (Pains,Burning):ti,ab,kw OR (Suffering,Physical): ti,ab,kw OR (Physical Suffering):ti,ab,kw OR (Physical Sufferings):ti,ab,kw OR (Sufferings,Physical):ti,ab,kw OR (Pain,Migratory):ti,ab,kw or (Migratory Pain): ti,ab,kw OR (Migratory Pains):ti,ab,kw OR (Pains,Migratory):ti,ab,kw OR (Pain, Radiating):ti,ab,kw OR (Pains,Radiating):ti,ab,kw OR (Radiating Pain):ti,ab,kw OR (Radiating Pains):ti,ab,kw OR (Pain,Splitting):ti,ab,kw OR (Pains,Splitting): ti,ab,kw OR (Splitting Pain):ti,ab,kw OR (Splitting Pains): ti,ab,kw OR (Ache):ti,ab,kw OR (Aches):ti,ab,kw OR (Pain,Crushing): ti,ab,kw OR (Pains,Crushing):ti,ab,kw OR (Crushing Pain):ti,ab,kw OR (Crushing Pains):ti,ab,kw |
|  | #4 (wrist-ankle acupuncture):ti,ab,kw OR (wrist-ankle needle):ti,ab,kw |
|  | #5 #1 AND #2 AND #3 AND #4 |
| Embase | #1 ‘random’:ab,ti OR ‘double-blind’:ab,ti OR ‘randomized controlled trail’:ab,ti OR ‘randomized’:ab,ti OR ‘placebo’:ab,ti |
|  | #2 ‘Neoplasms’:ab,ti OR ‘Neoplasia’:ab,ti OR ‘Neoplasias’:ab,ti OR ‘Neoplasm’:ab, ti OR ‘Tumors’:ab,ti OR ‘Tumor’:ab,ti OR ‘carcinoma’:ab,ti OR ‘Cancer’:ab,ti OR ‘Cancers’:ab,ti OR ‘Malignancy’:ab,ti OR ‘Malignancies’:ab,ti OR ‘Malignant Neoplasms’:ab,ti OR ‘Malignant Neoplasm’:ab,ti OR ‘Neoplasm,Malignant’:ab,ti OR ‘Neoplasms, Malignant’:ab,ti OR ‘Benign Neoplasms’:ab,ti OR ‘Neoplasms, Benign’:ab ti OR ‘Benign Neoplasm’:ab ti OR ‘Neoplasm,Benign’:ab,ti |
|  | #3 ‘Pain’:ab,ti OR ‘Pain,Burning’:ab,ti OR ‘Burning Pain’:ab,ti OR ‘Burning Pains’:ab,ti OR ‘Pains,Burning’:ab,ti OR ‘Suffering, Physical’:ab,ti OR ‘Physical Suffering’:ab,ti OR ‘Physical Sufferings’:ab,ti OR ‘Sufferings,Physical’:ab,ti OR ‘Pain, Migratory’:ab,ti OR ‘Migratory Pain’:ab,ti OR ‘Migratory Pains’:ab,ti OR ‘Pains,Migratory’:ab,ti OR ‘Pain, Radiating’:ab,ti OR ‘Pains,Radiating’:ab,ti OR ‘Radiating Pain’:ab,ti OR ‘Radiating Pains’:ab,ti OR ‘Pain,Splitting’:ab,ti OR ‘Pains, Splitting’:ab ti OR ‘Splitting Pain’:ab ti OR ‘Splitting Pains’:ab,ti OR ‘Ache’:ab,ti OR ‘Aches’:ab,ti OR ‘Pain,Crushing’:ab,ti OR ‘Crushing Pain’:ab,ti OR ‘Crushing Pains’:ab ti OR ‘Pains,Crushing’:ab ti |
|  | #4 ‘wrist-ankle acupuncture’:ab,ti OR ‘wrist-ankle needle’:ab,ti |
|  | #5 #1 AND #2 AND #3 AND #4 |
